# Bacteriotherapy

**Published:** 1887-02

**Authors:** 


BACTERIOTHERAPY.—Dr. Vineta-Bellaserra publishes in *La Revista de Ciencias Medicas* the results of experiments on the treatment of lupus vulgaris or tuberculosis by means of bacterium termo. Cantani used inhalations of bacterium termo in the treatment of phthisis, and as it is now generally admitted that the bacillus tuberculosis is also found in tissues affected with lupus, the writer considered this the best means of determining whether bacteriotherapy was practicable. Numerous trials were made and although the applications temporarily relieved pain, no permanent beneficial results were obtained.

---

IT is claimed by Dr. M. Naudin, that a drug has been discovered which is an infallible cure for consumption. This is the plant *Mutisia Viciæfolia*, which is indigenous in Bolivia. It has long been used by the natives in the treatment of all forms of respiratory diseases, and was probably employed by the Incas themselves. Dr. Sacc has sent the seeds to France where they have been planted in Le Jardin des Plantes and also some of the fluid extract to several European hospitals, so that we may expect a more accurate knowledge of the drug in the near future.

---

COLLIN, a French veterinarian, in a recent paper on animal obstetrics, cites the following facts, which may be of interest to medical men: "The period of gestation for individuals of the same species varies, but it is found that the duration of pregnancy is the same in the different gestations of the same animal. These facts are well known to stock-raisers, and they can estimate exactly the time of delivery of their mares and cows, excepting in the cases of primiparæ. The duration of pregnancy is shorter for female fœtuses than for male."

---

M. PAUL LEROY-BEAULIEN gives the following statistics of the consumption of tobacco in Europe. The rate per 1000 inhabitants is, according to him, as follows: Spain, 110 lbs.; Italy, 128 lbs.; Great Britain, 138 lbs.; Russia, 182 lbs.; Hungary, 207 lbs.; France, 210 lbs.; Denmark, 224 lbs.; Norway, 229 lbs.; Austria, 273 lbs.; Germany, 336 lbs.; Holland, 448 lbs.; Belgium, 560 lbs. In other words, the average Dutchman and Flemming use four or five times as much tobacco as the average Spaniard.

PROF. PAJOT, the distinguished French accoucheur, Tuesday, Dec. 20, 1886, resigned the Chair of Obstetrics in the Paris University, and also from active service in the hospitals where he has, for forty-five years, given clinical instructions. His pupils, old and young, combined in expressing their gratitude to their old teacher, and as a token of esteem presented him with a marble bust of himself, executed by the sculptor Charpentier.

---

DR. S. KALOMIN, director of the surgical clinic of St. Petersburg, Russia, suicided on account of the fatal termination of an operation performed at the request of the patient. His colleagues observed his great depression and assured him that the operation was skillfully performed, but to no purpose as the sequel proved.

---

THE birth-rate of Buffalo for 1886 was thirty-one and a halt per 1000, while in the neighboring village of Rochester it was but sixteen and two-thirds per 1000. There is some grave defect either in the vital statistics or in the fecundity of Rochester people.

---

THE widow of the late Dr. Von Gudden who lost his life while caring for the insane king of Bavaria has received from the Bavarian government a gift of $50,000. This is a God-send to her as the lamented doctor was blessed with eleven children, who are all living.

